# Recent Research on Different Parts and Extracts of *Opuntia dillenii* and Its Bioactive Components, Functional Properties, and Applications

**DOI:** 10.3390/nu15132962

**Published:** 2023-06-29

**Authors:** Wen-Chien Lu, Chien-Shan Chiu, Yung-Jia Chan, Amanda Tresiliana Mulio, Po-Hsien Li

**Affiliations:** 1 Department of Food and Beverage Management, Chung-Jen Junior College of Nursing, Health Sciences and Management, Chia-Yi City 60077, Taiwan; 2 Department of Dermatology, Taichung Veterans General Hospital, Taichung City 40705, Taiwan; 3 College of Biotechnology and Bioresources, Da-Yeh University, Changhua 51591, Taiwan; 4 Department of Food and Nutrition, Providence University, Taichung City 43301, Taiwan

**Keywords:** *Opuntia dillenii*, bioactive compositions, functional properties, applications

## Abstract

*Opuntia dillenii* (*O. dillenii*) is a plant belonging to the Cactaceae family that is abundant in tropical and subtropical regions worldwide. *O. dillenii* is consumed as a local delicacy and has no other current use. To understand the nutritional value of *O. dillenii* in human health and its application in the food, cosmetic, and drug industries, this review summarizes information on the chemical compounds (pure α-pyrone compounds, flavonoids, phenolic acids, polysaccharides, minerals, fatty acids, and betalains) and biological properties (anti-diabetic, anti-hyperglycemic, antihyperlipidemic, anti-atherosclerotic, anti-inflammatory, analgesic, antimicrobial, antifungal, antiviral, anti-spermatogenic, anticancer, antilarval, anti-angiogenic, and antioxidant) of extracts from each part of the plant (fruit juice, fruit peel, cladode, and seeds) (aqueous, ethanolic, and methanolic), and seed oil. In addition, data related to the recent applications of *O. dillenii* in various industries (e.g., edible coatings, food supplements, cosmetics, nanoparticles, and wastewater treatment) are provided.

## 1. Introduction

*Opuntia dillenii* (Cactaceae), also known as the prickly pear or pear bush, is a succulent shrub with a height of approximately 1–1.8 m [1,2]. *O. dillenii* is widely grown in tropical and subtropical regions worldwide as well as in desert and semi-desert areas, including Taiwan and southern China [3,4]. In Taiwan, *O. dillenii* is known as Xian Ren Zhang and is abundant on Penghu Island [5]. The local people on Penghu Island serve *O. dillenii* fruit in ice cream, pastry filling, beverages, and other desserts. As a traditional medicine, *O. dillenii* fruit has been used to treat gonorrhea and control spasmodic cough, whereas its leaves have been used to treat ophthalmia as well as to allay heat and inflammation [6]. The flowers of *O. dillenii* are yellowish-orange to lemon-yellow, and the flowering period is from April to July [7]. The stems are green, branched, flat, and fleshy and are known as cladodes. The leaves are tiny and shaped like cylindrical spines; the fruits are green but turn red when mature; and the cladode and fruits have glochids on their surfaces [2]. 

Many studies have focused on the pharmacological properties of *O. dillenii* because of the various bioactive compounds derived from its different parts. *O. dillenii* flowers have anti-inflammatory, analgesic, antioxidant, antiviral, and antimicrobial effects [8,9]. The fresh fruit of *O. dillenii* has antihyperglycemic effects, and its lyophilized aqueous fruit extract has anti-inflammatory properties [10,11]. The cladodes of *O. dillenii* possess anti-inflammatory properties by inhibiting arachidonic acid metabolites and cytokines and have potent hypotensive and antihyperglycemic effects [12,13,14]. *O. dillenii* seed oil has antidiabetogenic and antihyperlipidemic activities that are correlated with phenolic content and antioxidant activity [15]. This review aims to summarize the reported phytochemical compounds in different parts of *O. dillenii*, their beneficial bioactive properties for human health, and their use in different industries.

## 2. Chemical Compounds of *Opuntia dillenii*

**Figure 1:**
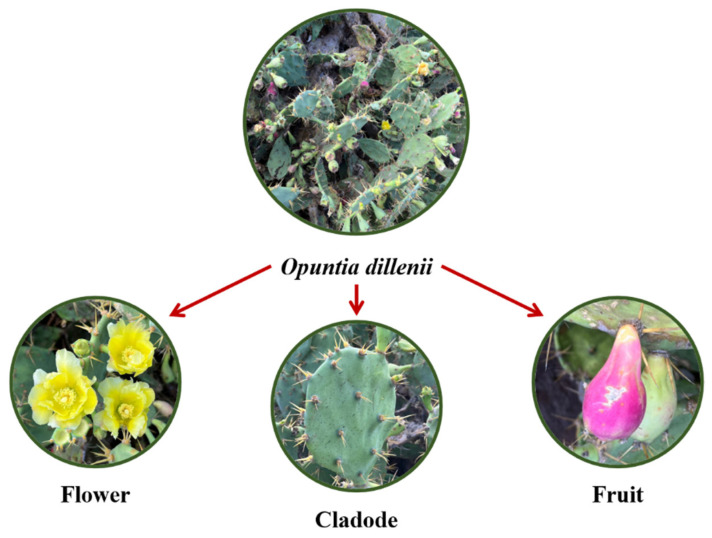
*Opuntia dillenii* on Penghu Island, Taiwan.

Different parts of *O. dilleni* contain varying amounts of compounds. An image of *O. dillenii* is shown in Figure 1, and Table 1 summarizes the chemical compounds in its fruits, mature stems, seeds, cladodes, and flowers.

### 2.1. Fruits

*O. dillenii* fruit contains more fiber, fat, ash, ascorbic acid, total phenolics, sodium, calcium, magnesium, and manganese than other species; hence, consuming *O. dillenii* fruit contributes to the intake of these nutrients [16]. Interestingly, consuming one serving (150 g) of *O. dillenii* fruit per day contributes to a high fiber intake (57% for the Spanish population), with a minimum recommended intake of 25 g [16]. Betalains are hydrophilic pigments that accumulate in the vacuoles of cells [24], and the fruit of *O. dillenii* is an edible source of betalains. A betalain content of 10.19 ± 0.13 mg/g was identified in the fruits of *O. dillenii*, where betacyanin (red-violet) is the major type [17]. Cold storage conditions (4 °C) are optimal for maintaining the initial redness, lightness, and betalain content of *O. dillenii* [25]. Specifically, the fruit pulp had a higher betalain content (444.77 mg/100 g f.w.), but the results of in vitro gastrointestinal digestion studies show that the bioaccessibility of betanin is only 22.9% [26].

The phenolic content of *O. dillenii* was more than two times higher (117 ± 10 mg/100 g) [16] than that of *Opuntia ficus indica*. Gómez-López et al. reported that the most abundant phenolic acids in *O. dillenii* whole fruits are protocatechuic acid derivatives (3.26 ± 0.18 mg/g dry weight) and piscidic acid (0.93 ± 0.00 mg/g dry) [17]. Protocatechuic acid has anti-inflammatory, antioxidant, anti-hyperglycemic, antibacterial, anti-aging, anti-angiogenic, antitumor, anti-asthma, anti-ulcer, antispasmodic, and neurological properties [27]. Lataief et al. reported that ethanol is a better solvent for extracting the *O. dillenii* fruit peel because more phenolic compounds are extracted, and quinic acid is the largest component (1437.03 μg/g) [18]. According to recent studies, quinic acid exhibits various biological activities, including antioxidant, antidiabetic, anticancer, antimicrobial, antiviral, anti-aging, protective, anti-nociceptive, and analgesic effects [28]. 

The whole fruit of *O. dillenii* also contains high levels of flavonoids, and glucoxyl-rhamnosyl-pentoside (IG2) is the most abundant (0.28 ± 0.00 mg/g dry weight). An ethanolic extract of *O. dillenii* fruit peel has 11 flavonoids (flavones, flavonols, flavanones, and flavanols), and rutin (11.42 μg/g), naringin (0.34 μg/g), and luteolin (6.16 μg/g) are the largest components. Rutin has many therapeutic properties owing to its potent antioxidant and anti-inflammatory activities [29].

### 2.2. Seeds

Ghazi et al. reported that *O. dillenii* seeds are rich in minerals; phosphorous is the major compound (970.15 mg/100 g), and the amount of zinc (78.26 mg/100 g) is higher than that of other trace elements, including iron, manganese, nickel, chromium, and copper [19]. The oil extracted from *O. dillenii* seeds by Soxhlet extraction and supercritical fluid extraction contained high levels of unsaturated fatty acids, with linoleic acid (≤62%) being the most abundant. In addition, trilinolein was the most abundant triacylglycerol (32–35%), and the reported phenol compounds were vanillin, 4-hydroxybenzaldehyde, vanillic acid, and γ-tocopherol at 81–89 mg/100 g [30]. Powdered *O. dillenii* seeds contain a high crude fiber content (52.78%) [31]. Despite their high nutritional content, ripe fruits of *O. dilleniid*, which contain 39.66% seeds, are considered by-products of the food processing industry and, hence, can be used to increase the functional and sensory properties of food products [31,32].

### 2.3. Cladodes

The methanolic extract of *O. dillenii* cladode contains the signature compounds opuntiol and opuntioside as anti-inflammatory agents, which are non-cytotoxic, non-hepatotoxic, non-nephrotoxic, and non-genotoxic [12,23]. In addition, ethanolic extracts of *O. dillenii* cladodes contain quinic acid, quercetin, rutin, luteolin, and cirsiliol as the major phenolic compounds. The major volatile compounds are n-hexadecanoic acid and stigmastan-3,5-diene [21]. Mucilage from the *O. dillenii* cladode exhibited pseudoplastic behavior and a good swelling index of 6.2%. Mucilage also contains neutral sugars, including galactose, rhamnose, and arabinose, and exhibits anti-obesity properties through lipase inhibition [22].

### 2.4. Flowers

The ethanolic extract of *O. dillenii* flowers contains three flavonoid glycosides (kaempferol 3-*O*-α-arabinoside, isorhamnetin-3-*O*-glucoside, and isorhamnetin-3-*O*-rutinoside) and shows potent anti-inflammatory and analgesic effects at a dose of 200 mg/kg [8]. The methanolic extract of *O. dillenii* flowers exhibited potent antioxidant abilities, with an IC_50_ of 58.7 μg/mL. This extract possesses strong antiviral activity against herpes simplex 1 and 2 and the vaccinia virus while exhibiting cytotoxicity above 100 μg/mL [9]. These studies indicate that *O. dillenii* flowers are suitable as natural antioxidants.

## 3. Biological Properties of *Opuntia dillenii*

**Figure 2:**
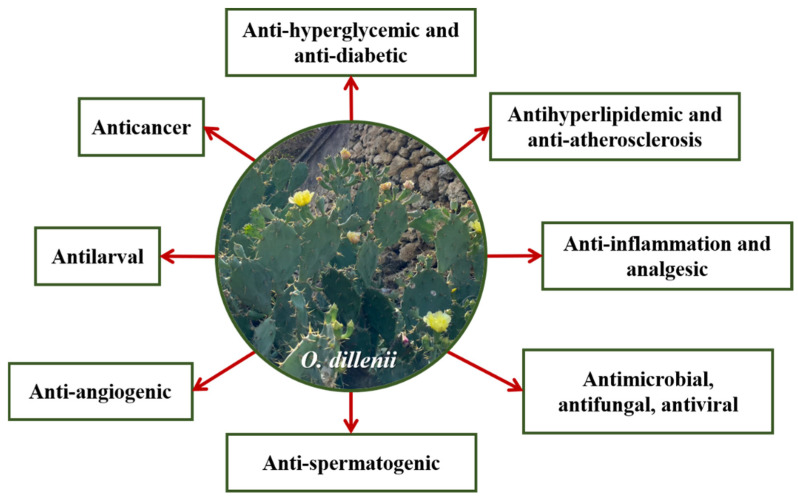
Functional properties of *Opuntia dillenii*.

As shown in Figure 2, a variety of *O. dillenii* biologically active compounds have been reported, including those with antidiabetic, antihyperglycemic, antihyperlipidemic, anti-atherosclerotic, anti-inflammatory, analgesic, antimicrobial, antifungal, antiviral, anti-spermatogenic, anticancer, antilarval, anti-angiogenic, and antioxidant properties.

### 3.1. Anti-Hyperglycemic and Anti-Diabetic Effects

Diabetes is among the top 10 causes of death in adults, affecting approximately 10% of the population and causing four million deaths globally in 2017 [33,34]. Half a billion people worldwide are living with diabetes, and this number is projected to increase by 25% by 2030 and 51% by 2045 [35]. Diabetes mellitus (DM) is a chronic metabolic disease characterized by high blood sugar levels, and more than 90% of DM cases are type 2 diabetes mellitus (T2DM) [36]. T2DM is associated with hyperglycemia and is caused by defective insulin secretion by pancreatic β-cells and the inability of insulin-sensitive tissues to respond to insulin owing to a complex network of pathological conditions [37]. Perfumi and Tacconi (1996) showed that drinking *O. dillenii* fruit juice has an anti-hyperglycemic effect by decreasing the intestinal absorption of glucose [10]. In addition, a water-soluble polysaccharide extracted from *O. dillenii* fruits has hypoglycemic and protective effects in streptozotocin (STZ)-induced diabetic rats by decreasing oxidative stress and preserving the integrity of pancreatic islets [38]. In addition to fruit juice, the oil extracted from *O. dilleni* seeds reduced blood glucose levels in STZ-induced rats and prevented diabetic effects in diabetic rats 30 days after administration [39]. The mechanism of the antidiabetic effect of *O. dillenii* seed oil involves inhibition of intestinal α-glucosidase and pancreatic α-amylase enzymes, as well as inhibition of intestinal absorption of D-glucose [40]. The ethanolic extracts of *O. dillenii* fruits, peels, and seeds contain naringin, catechin, and kaempferol, which are antidiabetic compounds [41]. ODP-Ia, a polysaccharide found in *O. dillenii* cladodes, exerts an antihyperglycemic effect by protecting the liver from peroxidation damage and by maintaining tissue function. Moreover, it aids the recovery of tissue function and improves the sensitivity of target cells to insulin in STZ-induced diabetic mice [14].

### 3.2. Antihyperlipidemic and Anti-Atherosclerosis Effects

Hyperlipidemia is an acquired condition or the result of a hereditary condition that refers to high fasting total cholesterol concentrations, elevated triglycerides, or both [42]. The *O. dillenii* cladode polysaccharide ODP-Ia is mainly composed of rhamnose, arabinose, galactose, glucose, and arabinuronic acid. ODP-Ia significantly decreases serum lipid levels and increases serum high-density lipoprotein cholesterol in hyperlipidemic Sprague-Dawley rats [43]. Atherosclerosis results from hyperlipidemia and lipid oxidation and is characterized by fatty plaques located in a central core that is found in all vascular systems [44]. Intraperitoneally injecting atherosclerotic rats with the *O. dillenii* polysaccharide (OPS) for 60 days showed that OPS has a significant anti-atherosclerotic effect by improving vasorelaxation in the thoracic aorta [45].

### 3.3. Anti-Inflammatory and Analgesic Effects

The fresh juice of *O. dillenii* has anti-inflammatory effects in rats with acetic-induced ulcerative colitis because of the presence of phenolic, flavonoid, and betalain compounds, which significantly reduce myeloperoxidase, malondialdehyde (MDA), and serum lactate dehydrogenase levels and enhance colonic levels of reduced glutathione [46]. In addition, the hydroalcoholic extract of *O. dillenii* fruit (100 and 200 mg/kg) exhibited hepatoprotective effects against Pb-induced liver toxicity in rats owing to its anti-inflammatory effect of reducing serum liver enzyme activities, MDA, and pathological scores, as well as increasing catalase levels [47]. Exposure to Cd seriously damages human health, leading to swelling of liver cells, exposure of the nucleus, central venous congestion, apoptosis, and inflammatory cell infiltration [48]. Mice with Cd-induced liver injury showed improved pathological indicators after the administration of OPS for 28 days (200 mg/kg) [48]. 

Paw edema induced by carrageenan in test animals is commonly used to determine anti-inflammatory effects [8,11,49]. The analgesic effect of a test sample on test animals can vary from electrical stimulation of the rat tail [49], acetic acid-induced writhing test [11,49], formalin-induced paw licking response [49], or hot-plate-induced jumping response [11,49]. Lyophilized *O. dillenii* crude aqueous fruit extract (100–400 mg/kg, injected intraperitoneally) exhibits analgesia associated with anti-inflammatory effects in carrageenan-induced paw edema in Sprague-Dawley rats and Swiss albino mice, and the results were obtained using the writhing and hot plate tests [11]. In addition to the fruit, the methanolic extract of the *O. dillenii* cladode containing opuntiol and opuntioside also showed analgesic effects by reducing peripheral and centrally mediated pain via an opioid-dependent and opioid-independent system in NMRI mice [49]. Siddiqui et al. (2016) reported that opuntioside has a better analgesic effect due to the presence of a sugar moiety in the α-pyrone ring, which causes faster absorption. They concluded that *O. dillenii* is a natural product with analgesic properties [49]. A 200 mg/kg dose of the alcoholic extract of *O. dillenii* flowers, containing the flavonoid glycosides kaempferol 3-*O*-α-arabinoside, isorhamnetin-3-*O*-glucoside, and isorhamnetin-3-*O*-rutinoside, showed the most potent anti-inflammatory and analgesic effects [8]. Fresh fruit pulp contains a higher betalain content, but when processed into jam, it contains only a few betalains [26].

### 3.4. Antimicrobial, Antifungal, and Antiviral Activities

Aqueous and ethanolic extracts of *O. dillenii* cladodes do not have antibacterial effects against gram-negative bacteria (*Salmonella enteritidis*, *Escherichia coli*, and *Klebsiella pneumoniae*). Instead, they are more sensitive to gram-positive bacteria (*Staphylococcus aureus* and *Micrococcus luteus*) [21]. In addition, the aqueous and ethanolic extracts of the *O. dillenii* cladode have antifungal activity against *Fusarium oxysporum*, with minimum inhibitory concentration (MIC) values of 2.34 and 4.68 mg/mL, respectively, and microbial fuel cells (MFC) of 18.75 and 75 mg/mL, respectively; there was no difference with the positive control cyclohexamide [21]. In another study, the methanolic extract of *O. dillenii* fruit was used for antibacterial screening and was reported to be active against 14 different bacterial strains at a concentration of 1000 μg/mL. This extract showed antifungal activity against six different fungal strains at concentrations of 500 and 1000 μg/mL [50]. The antibacterial activity of *O. dillenii* aqueous extracts was tested on six bacterial strains and compared to the standard antibiotic chloramphenicol, and the results showed that chloramphenicol had a significantly higher potential against all tested bacteria (MIC 0.63–2.5 mg/mL and 1.3–20 μg/mL, respectively) [51]. The seeds of *O. dillenii* from Nador extracted with ethyl acetate, ethanol, and water have antifungal activity against six fungal species with MIC values ranging from 0.16 to 2.5 mg/mL [51]. Interestingly, Katanić et al. found that the aqueous extract of *O. dillenii* fruits from Nador has a lower MIC value toward *K. pneumoniae* at 0.31 mg/mL compared to the aqueous extract of *O. dillenii* cladodes, with a MIC of 18.75 mg/mL [21,51]. Kumar et al. (2014) demonstrated that a methanolic extract of *O. dillenii* flowers has significant anti-herpes simplex types 1 and 2 (EC50 25 μg/mL and 20 μg/mL, respectively) and moderate antiviral activities against the vaccinia virus (EC50 100 μg/mL) [9].

### 3.5. Anti-Spermatogenic Activity

In 2002, Gupta et al. reported that the weights of the testes, epididymides, seminal vesicles, and venal prostate decreased significantly, and the production of spermatids decreased along with a reduced population of preleptotene spermatocytes, spermatogonia, and secondary spermatocytes after oral administration of an *O. dillenii* phylloclade methanolic extract (250 mg/kg) for 60 days in male Wistar rats [52]. Bajaj and Gupta (2012) treated male Wistar rats orally with a 100% methanolic extract of *O. dilleni* phylloclade (50 mg/kg) for 30 days and showed significantly reduced testosterone levels as well as significantly decreased epididymal sperm counts and motility.

### 3.6. Anticancer Activity

The ethanolic extract of *O. dillenii* cladodes has potent dose-dependent (100–500 μg/mL) anticancer activity against the HeLa cancer cell line compared to two other plants of the Cactaceae family, *Cereus pterogonus* and *Acanthocereus tetragonus* [53]. The ethanolic extract of *O. dillenii* cladodes has also been reported to be active against two human cancer cell lines (Caco-2 and K-562), with Caco-2 (colon cancer cell lines) being more sensitive than K-562 (lymphoblast cells) [21]. The effects of *O. dillenii* extract (fruit juice, skin, and seeds) on cell viability were tested in three human cancer cell lines derived from the colon (LoVo), liver (HepG2), and breast (MCF-7). None of the extracts were toxic to hepatic cancer cells, but the IC_50_ values in colon and breast cancer cells were determined only for the ethanolic seed extract [51]. An *O. dillenii* cladode extract tested on B16-F10 murine melanoma cells promoted wound healing, and 20 g/L *O. dillenii* extract significantly suppressed IBMX-induced melanogenesis, triggered cell proliferation, and decreased the mRNA expression of vascular endothelial growth factor and insulin-like growth factor-1, which prevented tumor proliferation and swelling [5].

### 3.7. Antilarval Activity

In addition to its potent anticancer activity, the ethanolic extract of *O. dillenii* cladodes also showed potent anti-larval activity in a larvicidal assay against fourth-instar larvae of *Aedes aegypti* in a dose-dependent manner (100–500 μg/mL) compared to two other Cactaceae family plants, *Cereus pterogonus* and *Acanthocereus tetragonus* [53].

### 3.8. Anti-Angiogenic Effects

The aqueous extract of *O. dillenii* has antiangiogenic potential by significantly reducing the total area and diameter of the primary, secondary, and tertiary blood vessels and capillary plexuses in a dose-dependent manner, which would be effective for cancer treatment [54].

### 3.9. Antioxidant Activity

In studies related to *O. dillenii*, DPPH is commonly used to measure antioxidant activity. The antioxidant activities of the different parts of *O. dillenii* are shown in Table 2. The polysaccharides extracted from *O. dillenii* cladodes have excellent antioxidant activity against DPPH radicals (8.80–58.44%) and good activity against hydroxyl radicals (0.84–45.69%) and superoxide radicals (5.22–43.71%) [3]. *O. dillenii* seed oil has DPPH scavenging activity with an IC_50_ value of 0.38 ± 0.08 mg/mL, and its phenolic content is correlated with antidiabetogenic and antihyperlipidemic activities [15]. In another study, the results of DPPH radical scavenging activity and the ascorbic acid test for *O. dillenii* juice (IC_50_ = 8.18 μL/mL) were higher than those of ascorbic acid alone (IC_50_ = 16.56 ± 0.019 μg/mL), making it suitable as a dietary complement or natural antioxidant [19]. The total antioxidant activity of the *O. dilleni* aqueous juice extract was the highest (830 ± 10 mg AAE/g dry extract) compared to other parts of the plant, whereas the ethanolic seed extract had the highest DPPH scavenging potential (IC_50_ = 45–63 μg/mL) and ABTS scavenging activity (IC_50_ = 88–130 μg/mL). However, the ethanolic extract of the seeds has a significantly lower antioxidant potential toward DPPH-scavenging radicals than reference compounds such as ascorbic acid, ellagic acid, and quercetin [51].

## 4. Applications for *Opuntia dillenii*

**Figure 3:**
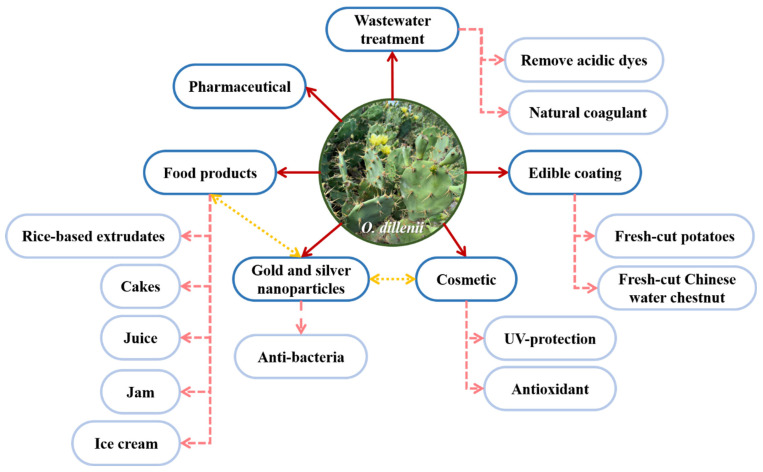
Applications of *Opuntia dillenii* in various fields.

As shown in Figure 3, *O. dillenii* has also been used in new product development, such as treating wastewater, preserving the shelf-life of freshly cut produce, as a cosmetic ingredient, producing gold and silver nanoparticles, and improving nutritional and sensory value during food product development.

### 4.1. Edible Coating

To extend the shelf life of fresh-cut potatoes, polysaccharides extracted from *O. dillenii* cladodes have been used to form edible coatings that suppress browning, microbial growth, and respiration rate, as well as inhibit weight loss and the formation of total sugars during storage at 5 °C for 5 days [55]. Another study reported that an edible coating made from OPS incorporated with glutathione extended the shelf life of fresh-cut Chinese water chestnuts stored at 3 °C for 10 days by suppressing the respiration rate, preventing weight loss, decreasing soluble solid content, and browning, as well as maintaining firmness [56]. 

### 4.2. Food Products

The seeds of *O. dillenii*, which are rich in fiber and contain high levels of antioxidants, have been incorporated into a rice-based extrude, which not only improves all sensory attributes in a sensory test but also enhances the fiber, phenolic, flavonoid, and antioxidant activities of the extrudates [31]. *O. dillenii* seed oil, which contains linoleic acid and exhibits strong antioxidant activity, has been used as a natural preservative in cake. No significant difference in peroxide value was detected between BHT-and *O. dillenii* seed oil-treated cakes [32]. *O. dillenii* fruit is used as a juice, and the physicochemical properties and bioactive compounds have been preserved by high hydrostatic pressure and thermal pasteurization [57]. The fruit pulp of *O. dillenii*, which has a low pH value, has been used in low-fat ice cream; the fruit pulp significantly increases the overrun of ice cream and slightly increases its melting rate [58]. Frozen whole fruit of *O. dillenii* has also been used to produce jam with very low phenolic and betalain content, which makes it difficult to use [26]. 

### 4.3. Cosmetics

*O. dillenii* is not commonly used in the cosmetics industry. Only one study has reported using an *O. dillenii* cladode extract to protect cells against ultraviolet light damage and inhibit melanin production, making it suitable for the development of skincare products [5]. In addition, Lataiefa et al. reported that an *O. dillenii* peel extract improves the antioxidant activity of cosmetic products owing to its total phenolic and total flavonoid contents, as well as its sensitivity toward *S. aureus*, *M. luteus*, and *P. catenulatum* [18]. 

### 4.4. Gold and Silver Nanoparticles

Nanoparticles made with noble metals, such as gold and silver, have been used in many fields, including cosmetics, and their synthesis can be carried out using green methods with plant extracts that contain phytochemical compounds as reducing agents and stabilizers [59]. Ahmed et al. reported that *O. dillenii* contains phytochemicals such as alkaloids, betacyanins, saponins, tannins, flavonoids, and phlobatannins. In addition, an *O. dillenii* extract alone with synthesized gold and silver nanoparticles exhibited a clear zone of inhibition toward bacteria, including *E. coli*, *S. aureus*, *K. pneumonia*, and *P. aeruginosa*, as well as fungal species, including *T. viride*, *C. albicans*, *C. krusei*, and *A. niger* [60].

### 4.5. Pharmaceutical Applications

*O. dillenii* fruit extract has been used as a traditional medicine to treat gastrointestinal and bronchial problems on the Canary Islands [11]. *O. dillenii* has been used to treat diabetes in Tunisia, Pakistan, and Canada [10,40,61,62]. The macerated leaf juice of *O. dillenii* has been used to maintain health and mental strength in Bangladesh [63]. *O. dillenii* is considered an important traditional medicine in China, Algeria, and Nigeria [64]. As described in Section 3, recent studies have reported *that O. dillenii* has biological properties (antidiabetic, antihyperglycemic, antihyperlipidemic, anti-atherosclerotic, anti-inflammatory, analgesic, antimicrobial, antifungal, antiviral, anti-spermatogenic, anticancer, antilarval, alti-angiogenic, and antioxidant), making it suitable for new drug development.

### 4.6. Wastewater Treatment

*O. dillenii* seeds have been reported to be adsorbents that significantly remove two acidic textile dyes, drimarene blue K_2_RL and eosin B, from large-scale aqueous media, where they can be recycled by desorbing with ethanol [65]. Nougbodé et al. (2013) reported that an aqueous *O. dillenii* solution made from stems clarifies very turbid surface water as a natural coagulant by suspending solids and eliminating color in the water when combined with lime [66].

## 5. Conclusions

*O. dillenii* is a valuable cactus plant because it contains important phytochemicals that promote human health. Phytochemical compounds derived from fruits, cladodes, and seeds consist of betalains, phenolic acids, flavonoids, minerals, fatty acids, polysaccharides, and α-pyrone compounds, which are responsible for various beneficial biological properties. Biological properties, such as antioxidant, anti-hyperglycemic, anti-diabetic, and anti-inflammatory properties, are commonly used as the main topic in research studies, while the remaining properties remain scarce. Interestingly, *O. dillenii* is not only applicable in food products, cosmetics, and pharmaceuticals but is also an environmentally friendly solution to treat wastewater. Hence, *O. dillenii* is a promising plant for the development of new products, owing to its wide application in various fields.

## Figures and Tables

**Table 1:** Chemical composition of different parts and extracts of *Opuntia dillenii*.

Part	Chemical Composition	Value	Ref.
Fruit	Fiber (%)	9.49 ± 1.51	[16]
	Protein (%)	0.52 ± 0.12	
	Fat (%)	0.71 ± 0.19	
	Ash (%)	0.437 ± 0.062	
	Acidity (g/100 g)	1.23 ± 0.272	
	Ascorbic acid (mg/100 g)	29.7 ± 2.95	
	Phenolics (mg/100 g)	117 ± 10	
	Na (mg/kg)	153 ± 162	
	K (mg/kg)	908 ± 251	
	Ca (mg/kg)	535 ± 187	
	Mg (mg/kg)	454 ± 102	
	Fe (mg/kg)	1.53 ± 0.31	
	Cu (mg/kg)	0.334 ± 0.054	
	Zn (mg/kg)	1.29 ± 0.49	
	Mn (mg/kg)	5.09 ± 3.80	
	Ni (mg/kg)	0.204 ± 0.082	
	Cr (mg/kg)	0.144 ± 0.036	
Whole fruit	Major betalains		[17]
	Betanin (mg/g)	2.75 ± 0.00	
	Isobetanin (mg/g)	1.60 ± 0.08	
	2′-*O*-apiosyl-4-*O*-phyllocactin (mg/g)	1.13 ± 0.08	
	5″-*O*-E-sinapoyl-2′-apyosil-phyllocactin (mg/g)	2.82 ± 0.02	
	Neobetanin (mg/g)	1.67 ± 0.00	
	Neobetanin isomer III v	0.23 ± 0.01	
	Total betalains (mg/g)	10.19 ± 0.13	
	Phenolic acid		
	Protocatechuic acid derivative (mg/g)	3.26 ± 0.18	
	Piscidic acid (mg/g)	0.93 ± 0.00	
	Total phenolic acids (mg/g)	4.19 ± 0.18	
	Major flavanoids		
	Quercetin-3-*O*-rhamnosyl-rutinoside (QG3) (mg/g)	0.03 ± 0.00	
	Quercetin hexose pentoside (QG2) (mg/g)	0.02 ± 0.00	
	Isorhamnetin glucosyl-rhamnosyl-pentoside (IG2) (mg/g)	0.28 ± 0.00	
Fruit peel (Aqueous extract)	Phenolic acids		[18]
	Quinic acid (μg/g)	118.95	
	Caffeic acid (μg/g)	0.041	
	P-coumaric acid (μg/g)	0.046	
	Trans ferulic acid (μg/g)	0.042	
	Flavonoids		
	Rutin (μg/g)	0.046	
	Naringin (μg/g)	0.007	
	Luteolin (μg/g)	0.014	
Fruit peel (Ethanolic extract)	Phenolic acids		[18]
	Quinic acid (μg/g)	1437.03	
	Protocatechuic acid (μg/g)	1	
	Caffeic acid (μg/g)	0.5	
	P-coumaric acid (μg/g)	1.27	
	Trans ferulic acid (μg/g)	2.4	
	Cinnamic acid (μg/g)	40.19	
	Flavonoids		
	Catechin (μg/g)	0.36	
	Hyperoside (μg/g)	0.92	
	Naringin (μg/g)	0.34	
	Quercetrin (μg/g)	0.36	
	Quercetin (μg/g)	9.27	
	Naringenin (μg/g)	0.29	
	Apeginin (μg/g)	0.095	
	Luteolin (μg/g)	6.16	
	Cirsiliol (μg/g)	7.18	
	Acacetin (μg/g)	0.19	
Fruit peel	Compounds		[18]
	4H-Pyran-4-one, 2,3-dihydro-3,5-dihydroxy-6-methyl- (area %)	2.21	
	2-Furancarboxaldehyde-5-(hydroxymethyl)- (area %)	32.91	
	Phenol, 2-methyl-5-(1-methylethyl)- (area %)	1.36	
	α-D-Glucopyranoside, α-D-glucopyranosyl (area %)	2.36	
	Phen-1,4-diol, 2,3-dimethyl-5-trifluoromethyl- (area %)	0.20	
	n-Hexadecanoic acid (area %)	3.52	
	Ethyl iso-allocholate (area %)	1.13	
	Vitamin E (area %)	1.11	
	β-Sitosterol (area %)	9.57	
Dry seeds	Macroelements		[19]
	Ca (mg/100 g)	408.28	
	Mg (mg/100 g)	240.30	
	Na (mg/100 g)	18.18	
	K (mg/100 g)	201.96	
	P (mg/100 g)	970.15	
	Trace elements		
	Fe (mg/100 g)	1.98	
	Cu (mg/100 g)	1.18	
	Zn (mg/100 g)	78.26	
	Mn (mg/100 g)	4.35	
	Cr (mg/100 g)	1.58	
	Ni (mg/100 g)	2.76	
Matured stems	Proximate composition		[20]
	Ash content (%)	3.33 ± 0.18	
	Moisture content (%)	11.20 ± 0.13	
	Crude protein (%)	11.60 ± 0.21	
	Fat (%)	4.42 ± 0.19	
	Crude fiber (%)	4.40 ± 0.06	
	Carbohydrate (%)	64 ± 0.14	
	Phytochemical analysis		
	Anthocyanin (μg/mL)	0.04 ± 0.02	
	Oxalate (μg/mL)	1.07 ± 0.01	
	Tanin (μg/mL)	13.62 ± 0.05	
	Rutin (μg/mL)	12.41 ± 0.26	
	Phenol (μg/mL)	4.66 ± 0.08	
	Lunamarine (μg/mL)	34.43 ± 0.35	
	Saponin (μg/mL)	118.08 ± 0.57	
	Sapogenin (μg/mL)	11.88 ± 0.09	
	Ribalinidine (μg/mL)	3.75 ± 0.09	
	Phytate (μg/mL)	0.18 ± 0.04	
	Kaempferol (μg/mL)	7.90 ± 0.06	
	Catechin (μg/mL)	44.90 ± 0.38	
	Fatty acid composition		
	Lauric acid (%)	7.78 ± 0.06	
	Myristic acid (%)	41.24 ± 0.55	
	Palmitic acid (%)	5.48 ± 0.0	
	Heptadecanoic acid (%)	11.32 ± 0.06	
	Stearic acid (%)	9.25 ± 0.03	
	Arachidic acid (%)	1.21 ± 0.06	
	Linoleic acid (%)	13.95 ± 0.02	
Cladode (Aqueous extract)	Phenolic acid and flavonoid		[21]
	Quinic acid (μg/g dw)	18.40	
	Protocatechuic acid (μg/g dw)	0.44	
	Caffeic acid (μg/g dw)	0.025	
	Syringic acid (μg/g dw)	0.003	
	P-coumaric acid (μg/g dw)	0.106	
	Naringin (μg/g dw)	0.01	
	Trans ferulic acid (μg/g dw)	0.16	
	Cinnamic acid (μg/g dw)	0.023	
	Compounds		
	Aromadendrene	Not mentioned	
	9,9-Dimethoxybicyclo [3.3.1] nona-2,4-dion	Not mentioned	
	6,7-Dimethylthieno [2,3-b] quinolin-3-ylamine	Not mentioned	
	2-Hydroxypentadecyl propanoate	Not mentioned	
	2-Hexyl-1-octanol	Not mentioned	
	1-Eicosene	Not mentioned	
Cladode (Ethanolic extract)	Phenolic acid and flavonoid		[21]
	Quinic acid (μg/g dw)	58.78	
	Gallic acid (μg/g dw)	0.72	
	Protocatechuic acid (μg/g dw)	0.38	
	Rutin (μg/g dw)	7.02	
	Hyperoside (μg/g dw)	0.24	
	P-coumaric acid (μg/g dw)	1.49	
	Naringin (μg/g dw)	0.15	
	Quercetrin (μg/g dw)	0.50	
	1,3-di-*O*-cafeoylquinic acid (μg/g dw)	0.77	
	Apegenin-7-*O*-glucoside (μg/g dw)	0.09	
	Trans ferulic acid (μg/g dw)	1.56	
	Salviolonic acid (μg/g dw)	0.28	
	Quercetin (μg/g dw)	11.5	
	Kampherol (μg/g dw)	0.21	
	Naringenin (μg/g dw)	0.16	
	Apeginin (μg/g dw)	0.10	
	Luteolin (μg/g dw)	4.93	
	Cirsiliol (μg/g dw)	3.22	
	Cirsilineol (μg/g dw)	0.15	
	Acacetin (μg/g dw)	0.29	
	Compounds		
	n-Hexadecanoic acid	Not mentioned	
	Oleic acid	Not mentioned	
	Octadecanoic acid	Not mentioned	
	Vitamin A	Not mentioned	
	4,6-Cholestadien-3β-ol	Not mentioned	
	Stigmastan-3,5-diene	Not mentioned	
	Retinol, acetate	Not mentioned	
	Vitamin E	Not mentioned	
	β-Sitosterol	Not mentioned	
Cladode	Neutral sugars		[22]
	Rhamnose (%)	15.70	
	Arabinose (%)	38.80	
	Xylose (%)	5.10	
	Galactose (%)	33.00	
	Glucose (%)	5.10	
	Uronic acid (%)	2.50	
Cladode (Methanolic extract)	Pure α-pyrone compounds		[23]
	Opuntiol (%)	1.04	
	Opuntioside (%)	5.34	
Flowers	Phytochemicals		[8]
	Flavonoids	Not mentioned	
	Tannins	Not mentioned	
	Alkaloids	Not mentioned	
	Anthraquinone glycosides	Not mentioned	
	Steroids	Not mentioned	

**Table 2:** Antioxidant activities of the different parts and extracts of *Opuntia dillenii*.

Sample	Method	Concentration	Inhibition Ratio	Ascorbic Acid	Ref.
Seed oil	DPPH	5 μL/mL	21.04 ± 0.071	21.02 ± 0.066	[19]
		10 μL/mL	26.19 ± 0.076	29.96 ± 0.091	
		15 μL/mL	29.81 ± 0.066	40.15 ± 0.060	
		20 μL/mL	42.60 ± 0.061	63.85 ± 0.064	
		IC_50_	27.21 ± 0.075	16.56 ± 0.019	
Whole fruit	ORAC	μmol Trolox eq./g dry weight	151.81 ± 1.86	-	[17]
Fruit juice	DPPH	5 μL/mL	39.15 ± 0.095	63.85 ± 0.064	[19]
		10 μL/mL	56.11 ± 0.080	63.85 ± 0.064	
		15 μL/mL	73.54 ± 0.164	63.85 ± 0.064	
		20 μL/mL	91.94 ± 0.031	63.85 ± 0.064	
		IC_50_	8.18 ± 0.010	16.56 ± 0.019	
Aqueous cladode extract	DPPH	IC_50_ mg/mL	0.54 ± 0.001	0.015 ± 0.00	[21]
	NO	IC_50_ mg/mL	0.15 ± 0.005	0.04 ± 0.001	
	FRAP	700 nm	1.39 ± 0.00	2.41 ± 0.00	
	TEAC	mM Trolox/g	0.46 ± 0.15	0.85 ± 0.02	
	TAC	mg AAE/g	60.44 ± 3.65	81.24 ± 0.14	
Ethanolic cladode extract	DPPH	IC_50_ mg/mL	0.60 ± 0.005	0.015 ± 0.00	[21]
	NO	IC_50_ mg/mL	0.06 ± 0.003	0.04 ± 0.001	
	FRAP	700 nm	1.97 ± 0.00	2.41 ± 0.00	
	TEAC	mM Trolox/g	0.59 ± 0.75	0.85 ± 0.02	
	TAC	mg AAE/g	62.99 ± 1.18	81.24 ± 0.14	
Seed oil	DPPH	IC_50_ mg/mL	0.38 ± 0.08	0.23 ± 0.01 μg/mL	[15]
Flower	DPPH	IC_50_ μg/mL	58.7 ± 0.00	1.2 ± 0.00	[9]

## Data Availability

Data is contained within the article.
